# Microglia play an important role in PRV infection-induced immune responses of the central nervous system

**DOI:** 10.1186/s12985-023-02118-8

**Published:** 2023-07-14

**Authors:** Xiuxiu Sun, Xinxin Jin, Xi Liu, Lumeng Wang, Li Li, Junjie Yang, Helong Feng, Zhengdan Lin, Cunlin Zhan, Wanpo Zhang, Changqin Gu, Xueying Hu, Xiaoli Liu, Guofu Cheng

**Affiliations:** 1 Division of Veterinary Pathology, College of Veterinary Medicine, Huazhong Agricultural University, Wuhan, China; 2 Henan Shengming Biotechnology Research, Xinxiang, China

**Keywords:** Pseudorabies virus, Central nervous system, Inflammation, Microglia

## Abstract

Pseudorabies virus (PRV) can infect multiple hosts and lead to fatal encephalitis. There is a significant increase in the number of microglia in the brain of animals infected with PRV. However, whether and how microglia contribute to central nervous system damage in PRV infection remain unknown. In the present study, we elucidated that PRV infection can cause more severe inflammatory cell infiltration, thicker and more numerous vessel sleeve walls, and more severe inflammatory responses in the brains of natural hosts (pigs) than in those of nonnatural hosts (mice). In a mice infection model, activated microglia restricted viral replication in the early stage of infection. Acute neuroinflammation caused by microglia hyperactivation at late-stage of infection. Furthermore, in vitro experiments revealed that microglia restricted viral replication and decreased viral infectivity. This may be associated with the phagocytic ability of microglia because we observed a significant increase in the expression of the membrane receptor TREM2 in microglia, which is closely related to phagocytosis, we observed that depletion of microglia exacerbated neurological symptoms, blood–brain barrier breakdown, and peripheral lymphocyte infiltration. Taken together, we revealed the dual role of microglia in protecting the host and neurons from PRV infection.

## Introduction

Pseudorabies virus (PRV), the etiological agent of Aujeszky's disease, belongs to the subfamily Alphaherpesvirinae of the family Herpesviridae [1–3]. The natural hosts and carriers of PRV are pigs. The clinical manifestations of PRV infection include fatal encephalitis, respiratory distress, stunted growth and development in growing and fattening pigs, reproductive failure in sows, and 100% mortality in newborn piglets [4]. PRV infection in piglets can result in neurological symptoms and a mortality rate of up to 100%. Studies have reported the presence of severe neurological symptoms in various nonnatural hosts with PRV infection [5–7]. PRV is a neurotropic virus that can infect the central nervous system (CNS) of animals via retrograde axonal transport, leading to nonsuppurative encephalitis, characterized by an increase in the number of microglia and infiltrating peripheral leukocytes and severe neuron loss in the brain [8, 9].

Microglia are the primary resident macrophages in the CNS and exhibit high dynamics and mobility [10, 11]. They play a key role in maintaining brain balance during early brain development and throughout life [12–14]. Microglia are activated in response to nerve injury, infection, or neurodegeneration, resulting in changes in their cell morphology and obvious proliferation [15]. A study has reported that microglia are powerful producers of type I interferons, proinflammatory cytokines (such as interleukin [16]-1β, IL-6, and tumor necrosis factor [TNF]-α), and chemokines (such as chemokine ligand 2 [CCL2], monocyte chemoattractant protein-1 [MCP-1], chemokine (C-X3-C motif) ligand 1 [CX3CL1]) in response to viral infection [17]. Cytokines are a class of protein molecules that promote the occurrence and development of inflammation [18, 19]. Moreover, microglia are recruited to eliminate infected neurons via phagocytosis to facilitate brain immunity [20]. Triggering receptor expressed on myeloid cells 2 (TREM2) is a type I transmembrane protein that is primarily expressed on microglia. Increasing evidence suggests the importance of TREM2 signaling in the regulation of microglial phagocytosis and the production of inflammatory cytokines. Furthermore, many studies have reported that several disease-associated TREM2 mutations decrease TREM2 distribution on the cell surface and impair microglial phagocytosis [21–23].

In the present study, we established a PRV infection model in Balb/C mice to elucidate the role of microglial activation in neuroinflammation.

## Results

### PRV infection in pigs induced microglial activation and neuroinflammation.

**Fig. 1:**
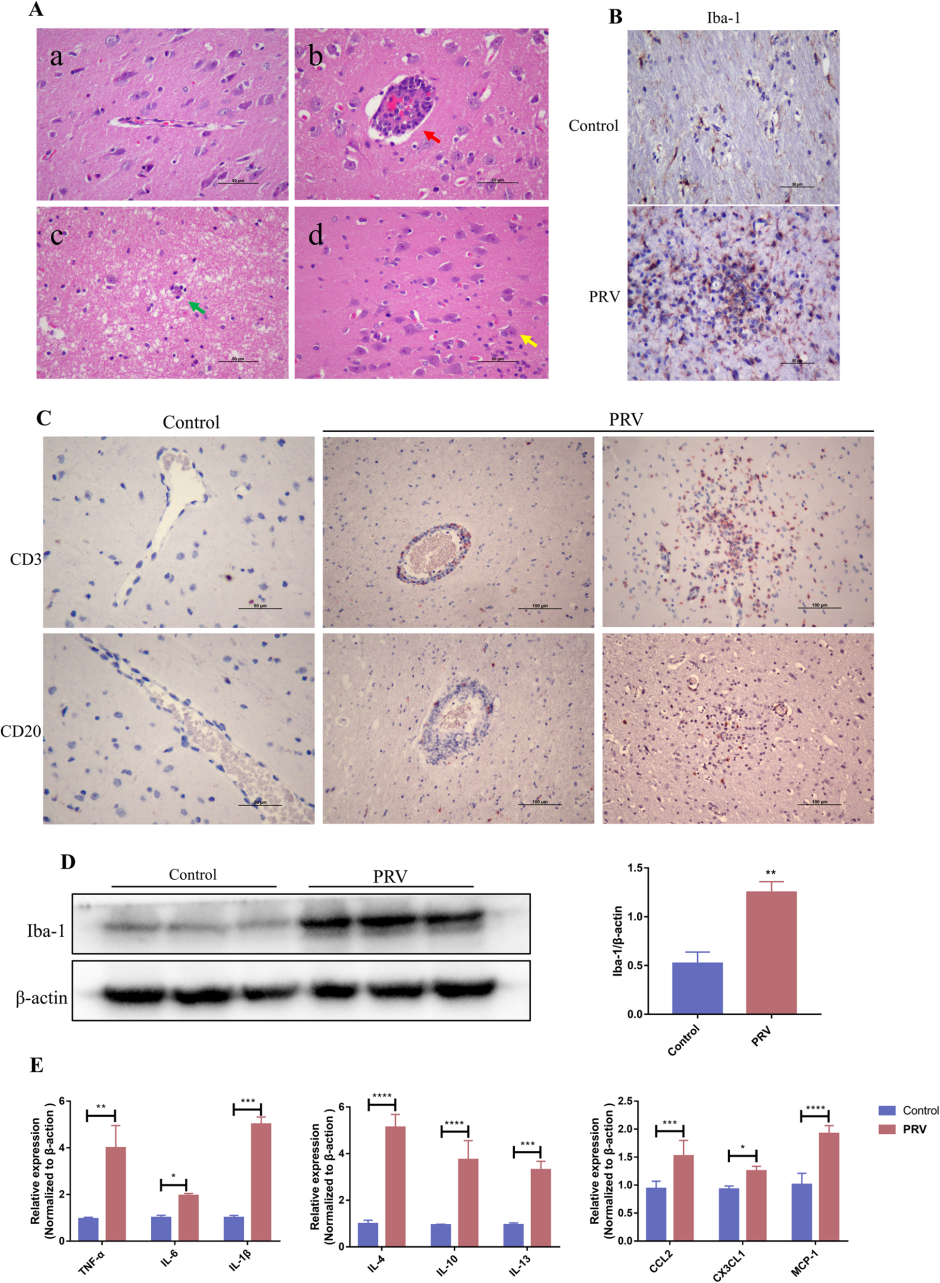
PRV infection induces microglial activation and neuroinflammation in pigs. Pig materials were obtained from Wu Bin’s laboratory at Huazhong Agricultural University. **A** HE staining showing histological changes in pig brains. Red arrow, vascular cuffs; green arrow, injured neurons surrounded by glial cells; and yellow arrow, glial nodules. **B** Immunohistochemical staining of Iba-1 in pig brain tissues. **C** Immunohistochemical staining of CD3 and CD20 in pig brain tissues. **D** Western blotting of Iba-1 and β-actin in pig brain tissues. **E** qRT-PCR showing cytokine expression (n = 3)

The rectal temperature of PRV-infected pigs was increased. Furthermore, they exhibited severe respiratory symptoms. Three pigs died: two on day 7 and one on day 10 post-infection. One pig had difficulty eating on day 4 post-infection [24]. Histological examination revealed typical pathological changes in viral encephalitis in brain tissues, including neuronal degeneration and necrosis (green arrow), glial nodule formation via astrocyte proliferation (yellow arrow), and a typical “perivascular cuffing” phenomenon characterized by a large number of infiltrating inflammatory cells surrounding the blood vessels (red arrow), which were visible throughout the brain (Fig. 1A). Immunohistochemical staining revealed that these inflammatory cells were mainly lymphocytes, with a high number of T lymphocytes (Fig. 1C). Iba-1 is an immunomarker for microglia. Immunohistochemical staining revealed that after PRV infection, microglia in the brain were significantly activated, with enlarged cell bodies and retracted processes, resembling amoeboid shapes (Fig. 1B). Western blotting revealed a significant increase in Iba-1 in the brain tissues of PRV-infected pigs (*P* < 0.01) (Fig. 1D). Furthermore, qRT-PCR analysis revealed that the mRNA expression of the proinflammatory cytokines TNF-α (*P* < 0.01), IL-6 (*P* < 0.05), and IL-1β (*P* < 0.001); anti-inflammatory cytokines IL-4 (*P* < 0.001), IL-10 (*P* < 0.001), and IL-13 (*P* < 0.001); and chemokines CCL2 (*P* < 0.001), CX3CL1 (*P* < 0.05), and MCP-1 (*P* < 0.001) was significantly increased in the brain of PRV-infected pigs (Fig. 1E).

### PRV infection in mice induces microglial activation and triggers neuroinflammation

**Fig. 2:**
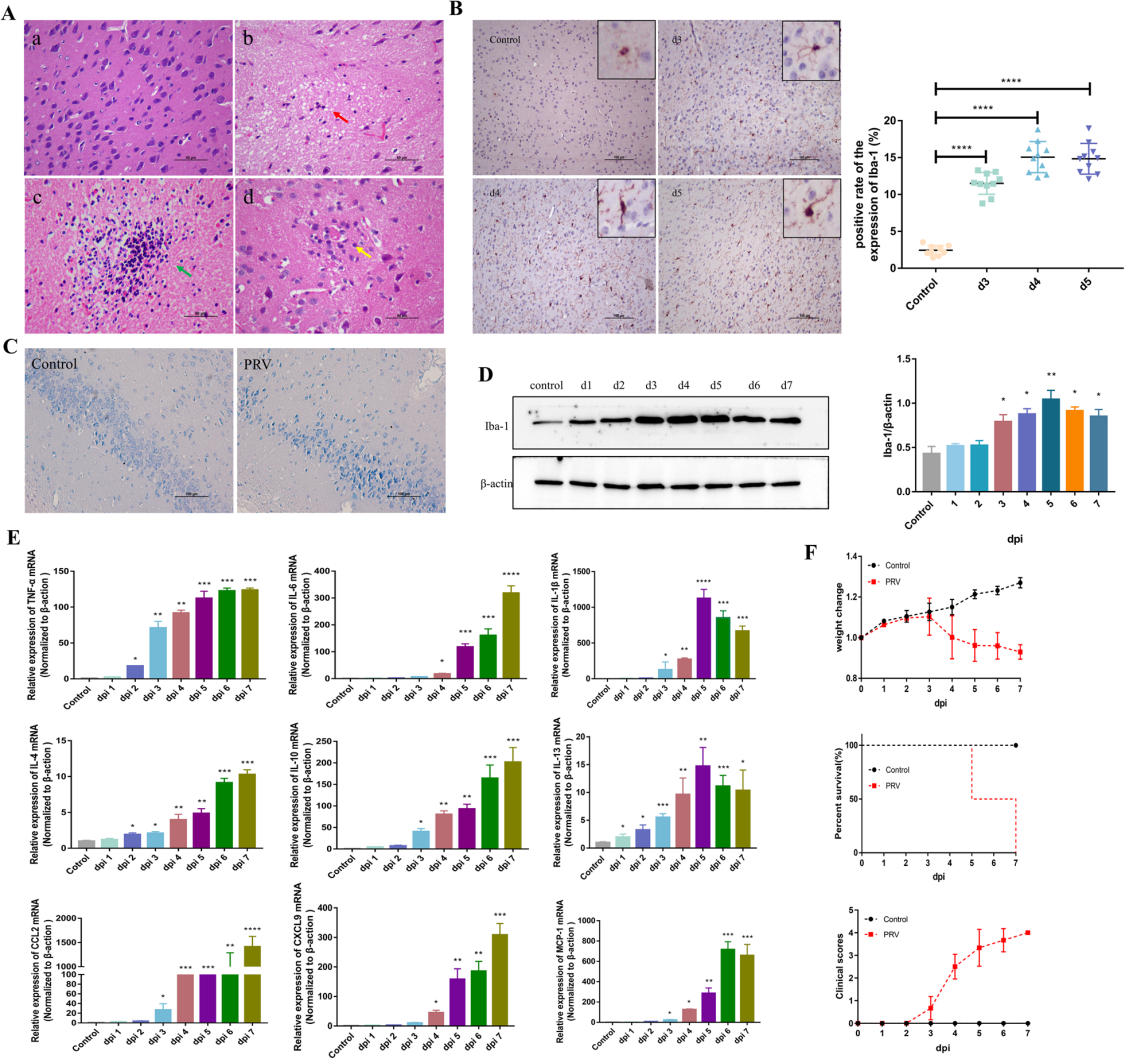
PRV infection induces microglial activation and neuroinflammation in mice. BALB/c mice were intranasally challenged with PRV (1 × 10^4^ TCID_50_). **A** HE staining showing histological changes in mice brains. Red arrow, vascular cuffs; green arrow, glial nodules; and yellow arrow, injured neurons surrounded by glial cells. **B** Immunohistochemical staining of Iba-1 in mice brain tissues. Iba1-positive cells were quantified in the right panels. Each column represents the mean ± SD for 10 fields in each group. Bars, 20 mm. **C** Nissl staining of mice brain tissues. **D** Western blotting of Iba-1 and β-actin in mice brain tissues. **E** qRT-PCR showing cytokine expression (n = 6). **F** Clinical manifestations of PRV-infected mice

We established a mouse PRV infection model and detected and analyzed immune indicators at different stages of the immune responses to elucidate the main immune response characteristics in the brain tissues of PRV-infected mice. PRV-infected mice exhibited decreased appetite, weight loss, and mild scratches on their faces on day 3. Furthermore, they refused to eat or drink, with continued weight loss, severe scratches on their faces, and significant neurological symptoms, and began to die on day 5. All mice died on day 7 (Fig. 2F). Histological observations revealed that the brains of PRV-infected mice exhibited obvious pathological changes related to viral encephalitis: neuronal degeneration and necrosis; visible nuclear condensation and rupture; enhanced acidophile of the cytoplasm; phagocytosis of dead neurons by macrophages, forming a “satellite phenomenon” (red arrow); glial cell proliferation, forming glial nodules (green arrow); and a large number of infiltrating lymphocytes and neutrophils around the blood vessels, forming a “vascular cuff” phenomenon (yellow arrow) (Fig. 2A). Nissl staining revealed that in the PRV infection group, the gaps became wider between the neurons, neurons became smaller, and staining became darker (Fig. 2C). Immunohistochemistry and western blotting were performed to detect microglia in the brain. The number of Iba-1-positive cells increased from day 3 in the PRV infection group (*P* < 0.001), exhibiting a “deformed parasite” state with enlarged cell bodies and decreased branches. On days 4 and 5 post-infection, the branches of microglia were further shortened and thickened and cell bodies were enlarged (Fig. 2B). Western blotting revealed that Iba-1 levels were increased (Fig. 2D). Immunohistochemistry and qPCR were performed to detect changes in the mRNA expression of cytokines in the brains of PRV-infected mice. On day 3, the expression of the proinflammatory cytokines TNF-α (*P* < 0.01), IL-6 (*P* < 0.01), and IL-1β (*P* < 0.05); anti-inflammatory cytokines IL-4 (*P* < 0.05), IL-10 (*P* < 0.05), and IL-13 (*P* < 0.05); and chemokines CCL2 (*P* < 0.05), CX3CL1 (*P* < 0.05), and MCP-1 (*P* < 0.05) were significantly upregulated; however, the increase in IL-4 and IL-13 expression was relatively low (Fig. 2E).

### Ki20227 decreased PRV-induced mouse neuroinflammation by depleting microglia

**Fig. 3:**
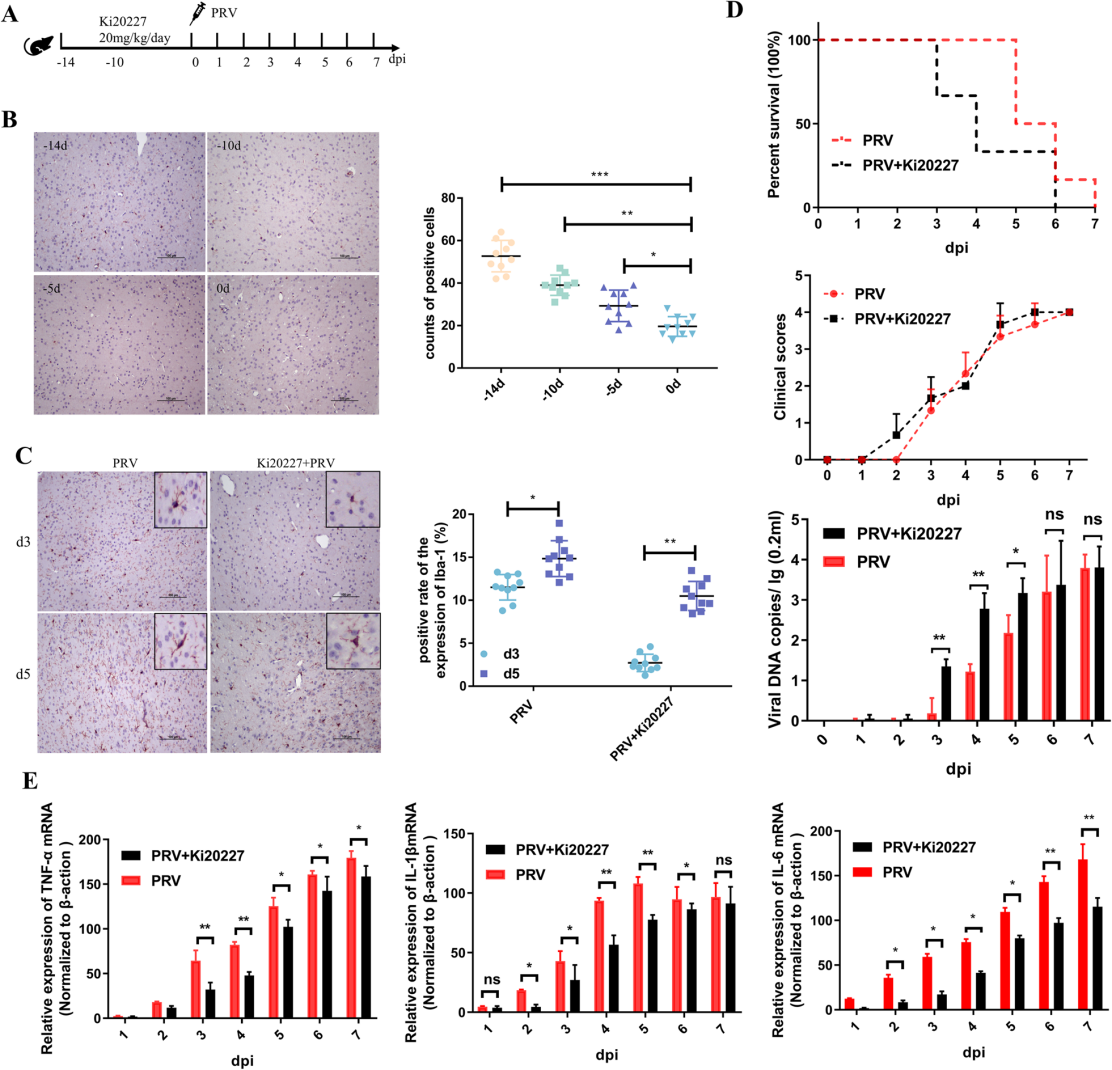
Ki20227 depletes microglia and exacerbates early neuroinflammation in mice. **A** Procedure of Ki20227 administration and PRV infection. **B** Microglial depletion was evaluated via immunostaining of Iba1. Iba1-positive cells were quantified in the right panels. Each column represents the mean ± SD for 10 fields in each group. Bars, 20 mm. **C** Immunohistochemical staining of Iba-1 in mice brain tissues. Iba1-positive cells were quantified in the right panels. Each column represents the mean ± SD for 10 fields in each group. Bars, 20 mm. **D** Clinical manifestations of the mice. **E** qRT-PCR showing cytokine expression (n = 6)

Previous studies have reported that Ki20227 can target and decrease microglia in the CNS by inhibiting colony-stimulating factor 1 receptor (CSF-1R) [25]. In this study, mice were orally administered PBS or Ki20227 (20 mg/kg/day) for 14 consecutive days before PRV infection to deplete microglia in the brain (Fig. 3A). The Ki20227-treated group exhibited clinical symptoms on day 2 post-infection, and all mice died by day 6. On the other hand, the PRV-infected control group exhibited clinical symptoms on day 3 post-infection, and all mice died by day 7. qPCR analysis of brain tissues revealed that PRV viral DNA expression was significantly higher in the Ki20227-treated group than in the PRV-infected control group (*P* < 0.01) on day 3 post-infection; however, the difference was not significant on day 6 (Fig. 3D). Immunohistochemical staining revealed that the efficiency of microglial depletion in the brain of Ki20227-treated mice was approximately 80% (*P* < 0.001) (Fig. 3A). Notably, the remaining microglia in the Ki20227-treated group were activated on day 3 post-infection, with rapid proliferation and activation of microglia on day 5 (Fig. 3C). To elucidate the role of microglia in PRV-induced nonpurulent encephalitis, qPCR was performed to detect the mRNA expression of cytokines in the brain. Ki20227 treatment significantly inhibited the expression of the proinflammatory cytokines TNF-α (*P* < 0.01), IL-6 (*P* < 0.01), and IL-1β (*P* < 0.05) on day 3 post-infection; however, no significant difference was observed in the expression of the Ki20227-treated and PRV-infected control groups on days 4 and 5. On the other hand, the expression of the chemokines CCL2 (*P* < 0.05), CX3CL1 (*P* < 0.05), and MCP-1 (*P* < 0.05) was significantly increased from day 3 post-infection. However, the expression of the anti-inflammatory cytokines IL-4 (*P* < 0.05), IL-10 (*P* < 0.05), and IL-13 (*P* < 0.05) was not significantly affected (Fig. 3E).

**Fig. 4:**
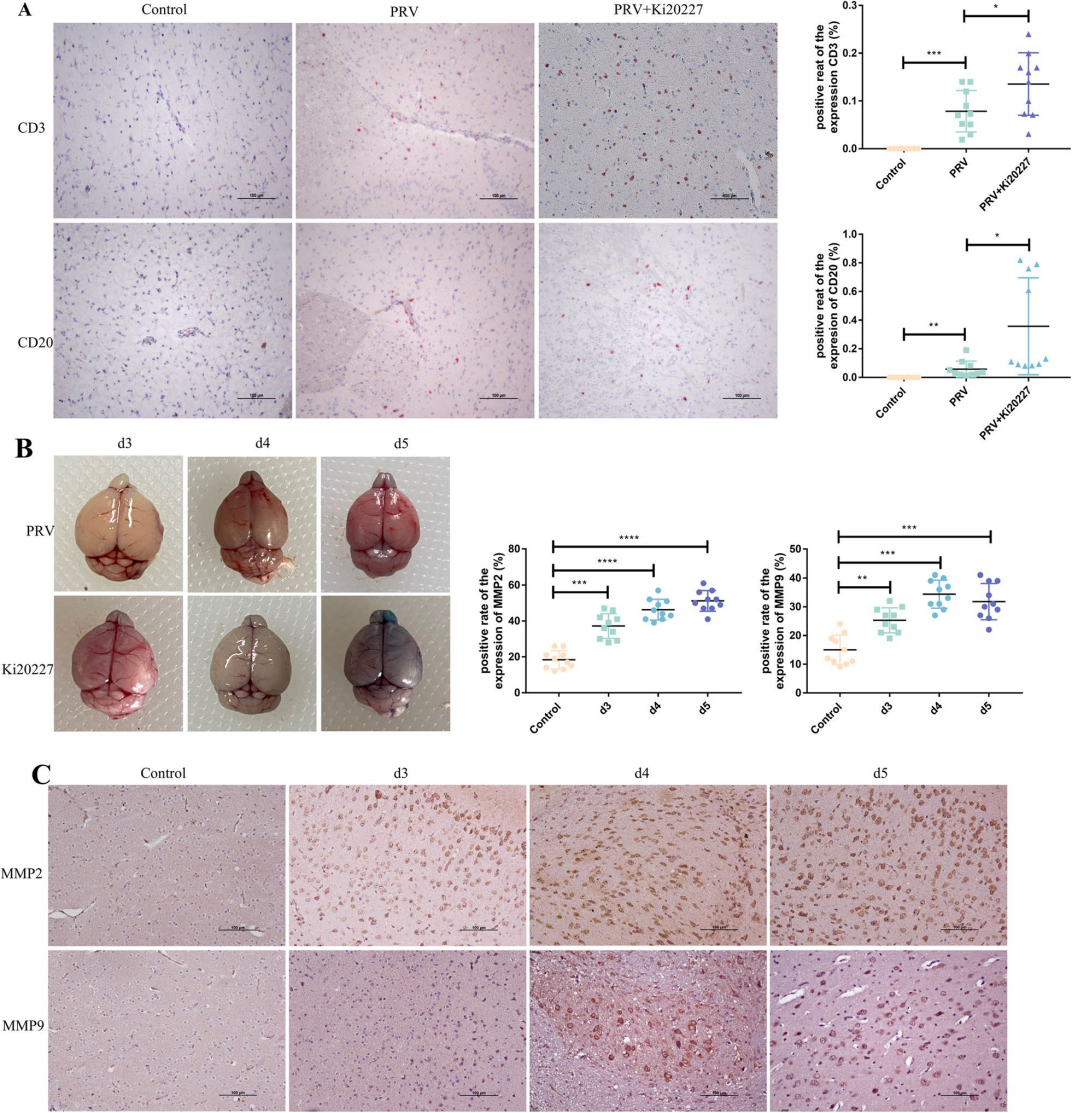
PRV infection promotes blood–brain barrier disruption and lymphocyte infiltration. **A** Immunohistochemical staining of CD3 and CD20 in mice brain tissues. Positive cells were quantified in the right panels. Each column represents the mean ± SD for 10 fields in each group. Bars, 20 mm. **B** Brain tissues stained with Evans blue. **C** Immunohistochemical staining of MMP2 and MMP9 in mice brain tissues. Positive cells were quantified in the right panels. Each column represents the mean ± SD for 10 fields in each group. Bars, 20 mm

There is often a positive correlation between immune cell infiltration and inflammatory reactions (16). To further confirm the proinflammatory role of microglia, we observed the effect of microglial depletion on cell infiltration induced by PRV infection in brain tissues. Immunohistochemical staining revealed that the number of CD3- (T lymphocyte marker) positive cells (*P* < 0.01) and CD20- (B lymphocyte marker) positive cells (*P* < 0.001) was significantly increased in the brain tissues of PRV-infected control mice, mainly distributed around the blood vessels. Furthermore, compared with PRV-infected control mice, the number of CD3-positive cells (*P* < 0.05) and CD20-positive cells (*P* < 0.05) was significantly increased in the brain tissues of infected mice treated with Ki20227 (Fig. 4A). Under normal conditions, no peripheral immune cells, such as lymphocytes and monocytes, are present in the brain. However, peripheral immune cells will enter the brain parenchyma with blood after damage to the blood–brain barrier. By injecting Evans blue solution via the tail vein, we observed changes in the permeability of the blood–brain barrier of PRV-infected mice. Furthermore, on day 4 post-infection, Evans blue began to get deposited in the olfactory bulb in the brain tissues of PRV-infected control mice. Nevertheless, the distribution of Evans blue did not significantly increase on day 5 post-infection. In the brain tissues of infected mice treated with Ki20227, Evans blue began to get deposited in the olfactory bulb on day 3 and was distributed throughout the brain tissue on day 5 (Fig. 4B). Immunohistochemical staining was performed to detect the expression of matrix metalloproteinases (MMP2 and MMP9) in the brain; the expression was significantly increased from day 3 post-infection (*P* < 0.01).

### BV2 cells engulfed and processed PRV

**Fig. 5:**
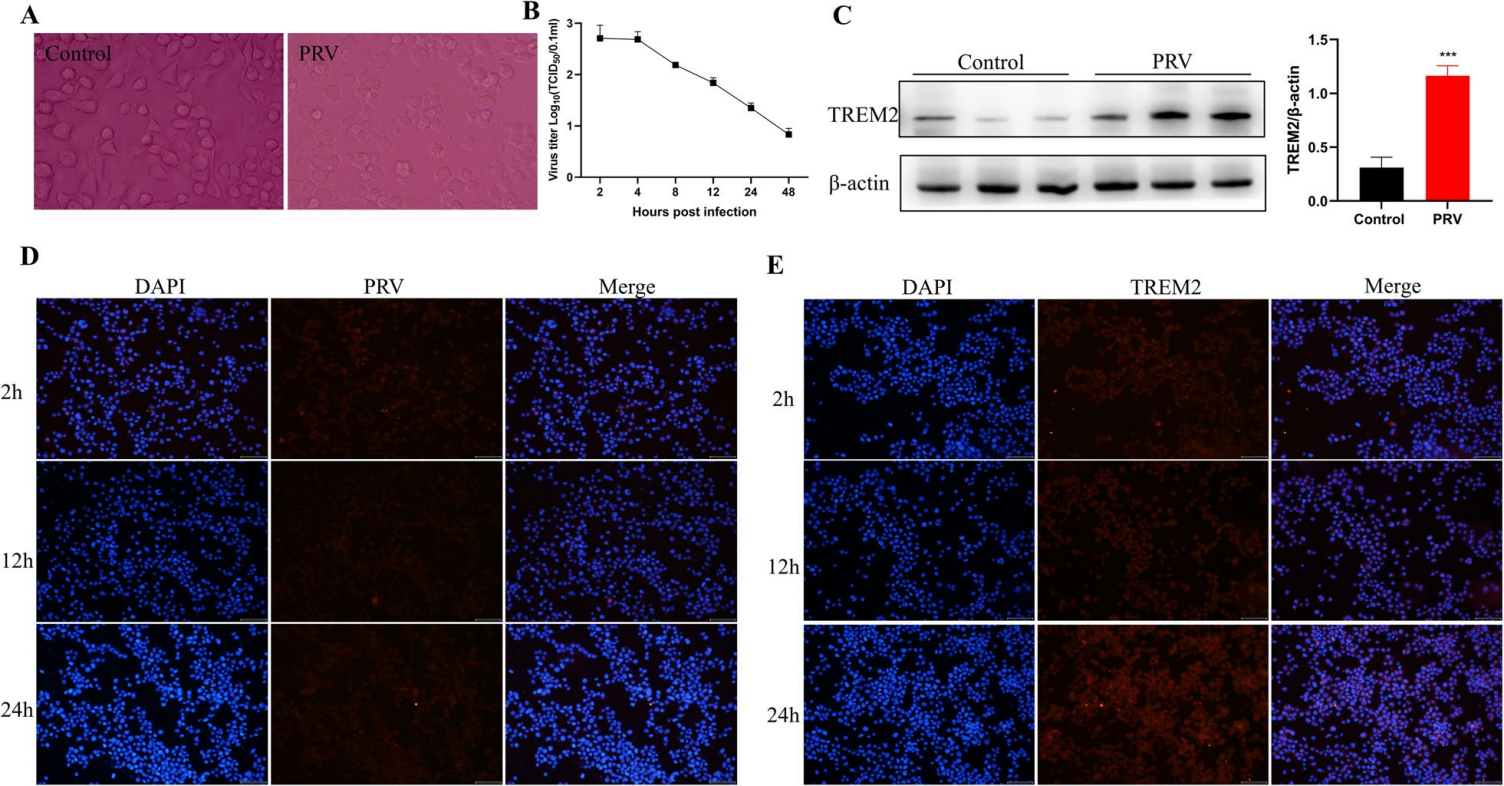
BV2 cells engulfed and processed PRV. **A** Optical inverted microscope showing the morphology of BV2 cells. **B** One-step growth curve. **C** Western blotting of TERM2 and β-actin in BV2 cells. **D** Immunofluorescence analysis of PRV-gE in BV2 cells. **E** Immunofluorescence analysis of TREM2 in BV2 cells

A previous study has reported that PRV replication significantly increases after treating microglia with Ki20227 [26]. Therefore, PRV was inoculated into BV2 cells. The refractive index of BV2 cells was increased, cells became round, and cell protrusions disappeared (Fig. 5A). Immunofluorescence analysis of PRV-gE protein levels in BV2 cells revealed that the positive signal of PRV-gE appeared in the cytoplasm at 2 h but disappeared at 24 h (Fig. 5D). Subsequently, we collected the cells and supernatants of PRV-infected BV2 cells at different time points (2, 4, 8, 12, 24, and 48 h) and inoculated them into PK-15 cells to determine the growth curve of the virus. PRV virus did not proliferate in BV2 cells (Fig. 5B). The engulfment ability of TREM2 protein in microglia is closely related. Immunofluorescence analysis revealed that PRV caused a significant increase in TREM2 protein levels in BV2 cells (Fig. 5C). Western blotting revealed the same trend in PRV-infected BV2 cells for 24 h.

### PRV induces BV2 cell activation

**Fig. 6:**
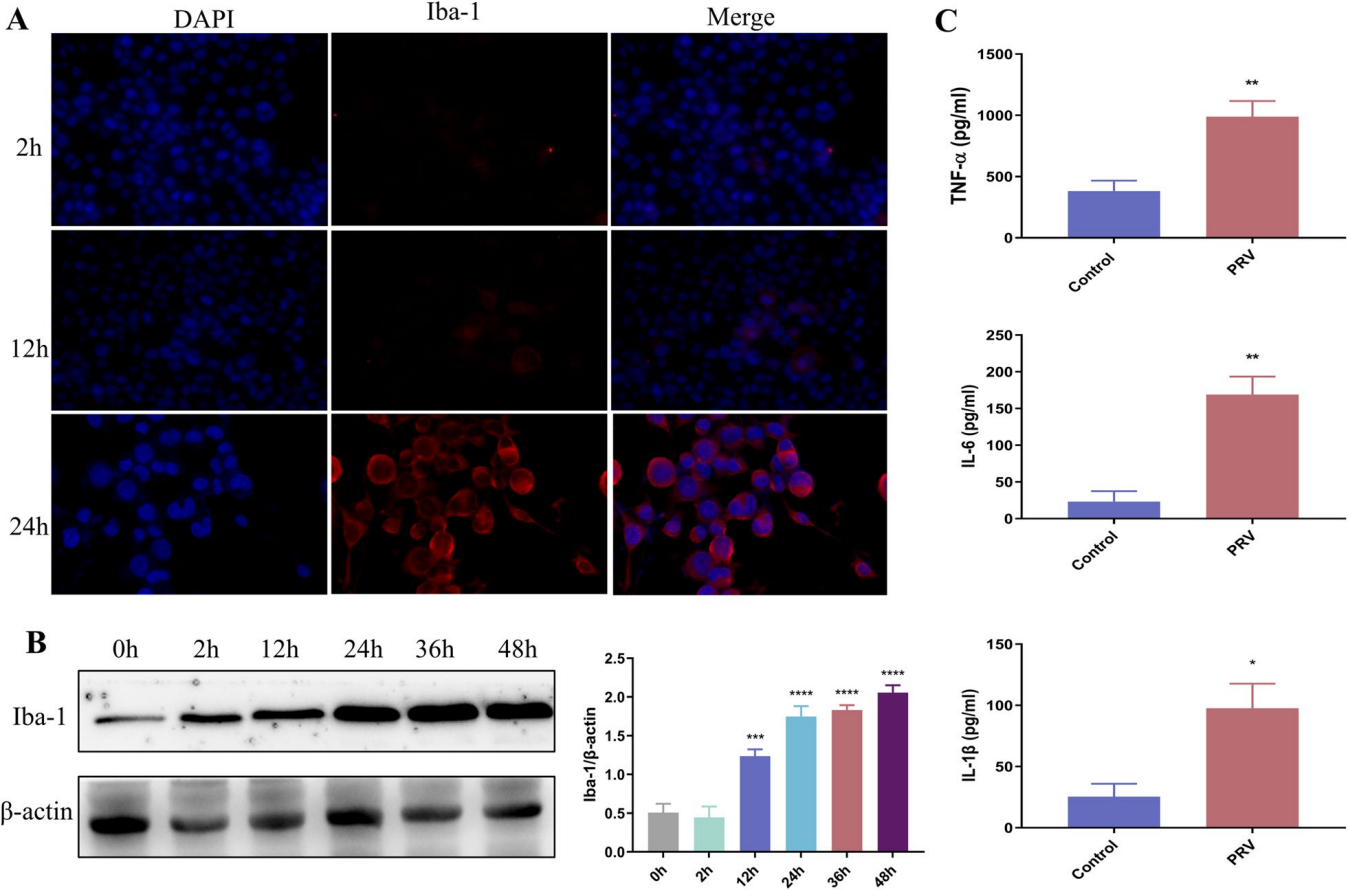
**A** Immunofluorescence analysis of Iba-1 in BV2 cells. **B** Western blotting of Iba-1 and β-actin in BV2 cells. **C** ELISA showing cytokine secretion (n = 3)

Immunofluorescence analysis was performed to detect BV2 cell activation. The number of Iba-1-positive cells was very low at 2 h; however, it began to increase at 12 h and was significantly increased at 24 h (Fig. 6A). Western blotting revealed the same trend in PRV-infected BV2 cells (Fig. 6B). ELISA was performed to detect inflammatory factors in the cell supernatant. The levels of TNF-α, IL-1β, and IL-6 were significantly increased (Fig. 6C).

## Discussion

The CNS has been regarded as an immune privilege for a long time because it cannot generate an immune response against allogeneic grafts [27]. Nevertheless, new evidence has prompted a re-evaluation of the CNS as not being an immune-privileged site but rather an “immunologically quiescent” one [28]. PRV is a well-known virus that can invade the nervous system. PRV infection in pigs is characterized by acute neurological symptoms and the possibility of developing nervous system disorders [29]. Several studies have verified that PRV-infected mice typically exhibit neurological infection of the CNS, accompanied by severe central nervous symptoms and a high mortality rate [30]. However, systematic studies on PRV-induced neuroinflammation are lacking. In the present study, we elucidated the neuroinflammation caused by PRV infection in pigs and used a PRV-infected mouse model to elucidate the role of activated microglia in neuroinflammation by altering the status and number of microglia.

We observed that PRV can cause severe neurological inflammation in both pigs and mice. Interestingly, the number of “vascular cuffs” in pig brain tissues was higher than that in mice brain tissues; furthermore, the number of infiltrating peripheral immune cells around the blood vessels was higher. This may be associated with the different courses of the two animals after infection. Mice died acutely after PRV infection with a course of only 7 days [31], whereas adult pigs had a longer course of PRV infection, providing more time for peripheral immune cells to react.

Damage to the blood–brain barrier is a prerequisite for the entry of peripheral immune cells into the injured site. West Nile virus infects the cells in the blood–brain barrier, resulting in considerable endothelium impairment, potent neuroinflammation, and immune cell recruitment [32]. Furthermore, after herpes simplex virus 1 infection, changes in blood–brain barrier integrity and permeability can lead to increased movement of viruses, immune cells, and/or cytokines into the brain parenchyma, resulting in more severe neuroinflammation and further brain damage [33, 34]. MMPs are encoded by multiple genes and belong to the zinc-dependent endopeptidase family. They are involved in the remodeling of the extracellular matrix. MMPs can cleave tight junction proteins, directly affecting the integrity of the blood–brain barrier [35, 36]. As a result, immunohistochemical staining was performed to assess the expression of MMP-2 and MMP-9. In this study, the expression of MMP-2 and MMP-9 was increased in the brain of pigs and mice, indicating that PRV infection damaged the blood–brain barrier.

After entering the brain tissue, PRV destroys the stability of the nervous system and rapidly activates microglia. Microglia secrete chemokines such as CCL2, CX3CL1, and MCP-1 and induce the infiltration of peripheral immune cells. This is a mutually reinforcing positive feedback regulation process. The infiltration of peripheral immune cells is essential for the immune responses of the CNS and plays a vital role in CNS infections [37]. An inflammatory response is generally caused by various inflammatory cytokines [38]. These peripheral immune cells then release inflammatory components. We observed the expression of the proinflammatory cytokines TNF-α, IL-6, and IL-1β; anti-inflammatory cytokines IL-4 (*P* < 0.05), IL-10, and IL-13; and chemokines CCL2, CX3CL1, and MCP-1, which were significantly increased in the brain tissue of pigs and mice. Furthermore, the expression of these cytokines was significantly increased in mouse brain tissues after the breakdown of the blood–brain barrier.

CSF-1R, a transmembrane tyrosine kinase, is involved in the proliferation and differentiation of microglia in the brain [39]. Ki20227, a CSF-1R antagonist, is widely used to deplete microglia in the CNS [25, 40]. In the present study, Ki20227 was administered to elucidate the role of microglial activation in PRV infection. We observed that PRV replication was relatively rapid in the brain tissues of Ki20227-treated mice treated in the early stages of infection; however, as the remaining microglia in the brain were activated, viral replication weakened. Studies have reported that microglia can engulf and degrade prions, thereby decreasing viral infection in the CNS [41, 42]. This is closely associated with the phagocytic ability of microglia against viruses, which was confirmed in subsequent cell experiments. These findings suggest that damage to the CNS is not the key factor for moderate microglial activation but may be related to PRV replication during the early stage of infection. Meanwhile, in Ki20227-treated mice, the blood–brain barrier was damaged earlier and more severely with more peripheral cell infiltration and a larger infiltration area. This may be related to the failure of microglia to interfere with early PRV replication.

In summary, we have provided evidence that microglia play an important role in PRV-induced neuroinflammation. Microglia can engulf and process early PRV replication and secrete cytokines to cope with local injury and chemotactic peripheral immune cell infiltration.

## Material and methods

### Virus

Virulent PRV was donated by Wu Bin’s laboratory at Huazhong Agricultural University.

### Animals

Pig materials were obtained from Wu Bin’s laboratory at Huazhong Agricultural University. Six-week-old female Balb/C mice were purchased from the Laboratory Animal Center of Huazhong Agricultural University (Wuhan, China). All mice were randomly divided into three groups, with six mice in each group: control group, intranasally infected with 20 µL of Dulbecco's modified Eagle medium, 10 µL per nostril; PRV infection group, intranasally infected with 20 µL of PRV viral suspension (1 × 10^4^ TCID_50_), 10 µL per nostril; and Ki20227 treatment group, Ki20227 (20 mg/kg/day) was orally administered for 14 consecutive days, followed by intranasal infection with 20 µL of PRV viral suspension (1 × 10^4^ TCID_50_), 10 µL per nostril. The clinical manifestations of the mice were observed and their weight was measured at the same time every day. At the end of the experiment, mouse brain tissues were collected for tissue sectioning and RNA and protein extraction. The study protocols were performed according to the Committee for Protection, Supervision, and Control of Experiments on Animals Guidelines of Huazhong Agricultural University (approval number: HZAUMO-2023–0080).

### Cell culture

Mouse microglia (BV2) were purchased from Warner Bio Co., Ltd. (Wuhan, China). BV2 cells were seeded into a 6-well plate and cultured for 24 h until 60%–80% confluency. Serum-free medium was added and 1 MOI PRV was inoculated. Cell samples were collected after culturing for 24 h.

### Detection of blood–brain barrier integrity and permeability

Mice were injected with 1% Evans blue (10 mL/kg) via the tail vein and allowed to freely move for 24 h. After mice were anesthetized with 0.5% pentobarbital sodium, they were perfused with 20 mL of pre-cooled phosphate-buffered saline (PBS) via the heart. Finally, brain tissues were isolated to observe the precipitation of Evans blue.

### Hematoxylin and eosin (HE) staining

After mice were euthanized, brain tissues were immediately removed and fixed with 4% paraformaldehyde for more than 24 h. Then, tissues were dehydrated using a series of procedures, embedded in paraffin blocks, cut into 3-µm sections, and stained with HE.

### Immunohistochemical staining

Brain tissue sections were deparaffinized with xylene and a gradient of alcohol and water. Then, heat retrieval was performed using sodium citrate buffer (pH 6) for 15 min. After blocking with a blocking reagent in the dark at room temperature for 10 min, the sections were incubated with goat serum at room temperature for 30 min. Then, the sections were incubated with the primary antibody overnight at 4 °C, followed by incubation with a biotinylated secondary antibody at room temperature for 30 min. Finally, immunohistochemical staining was performed using solid 3,3-diaminobenzidine.

### Immunofluorescence analysis

Paraffin-embedded brain tissue sections were deparaffinized with xylene and a gradient of alcohol and water. Then, heat retrieval was performed with sodium citrate buffer (pH 6) for 15 min. Sections were then blocked with goat serum at room temperature for 30 min, incubated with primary antibody overnight at 4 °C, and incubated with fluorescent secondary antibody at room temperature for 1 h. Thereafter, sections were stained with 4′,6-diamidino-2-phenylindole (DAPI) for 10 min. For cultured cells, they were fixed with 4% paraformaldehyde for 30 min, permeabilized with 1% Triton X-100 for 10 min, blocked with goat serum for 30 min, incubated with primary antibody overnight at 4 °C, and then incubated with fluorescent secondary antibody at room temperature for 1 h. Finally, cells were stained with DAPI for 10 min. The samples were observed and photographed under a fluorescence microscope.

### Quantitative real-time polymerase chain reaction (qRT-PCR) analysis

Total RNA was extracted from mouse brain tissues or cells using Trizol. cDNA was synthesized using a reverse transcription kit. The SYBRGreen reagent and StepOne real-time system were used for qRT-PCR. Table 1 presents the primers used for qRT-PCR. The cycling conditions were as follows: 3 min at 95 °C, followed by 40 cycles of 10 s at 95 °C, 30 s at 54 °C, and 30 s at 72 °C. Data analysis was performed using the 2^−ΔΔCt^ method.

**Table 1:** Primers for quantitative real-time polymerase chain reaction

β-actin Forward:	CACTGCCGCATCCTCTTCCTCCC
β-actin Reverse:	CAATAGTGATGACCTGGCCGT
TNF-α Forward:	TGGCCTCCCTCTCATCAGTT
TNF-α Reverse:	TTGAGATCCATGCCGTTGGC
IL-6 Forward:	CTTCTTGGGACTGATGCTGG
IL-6 Reverse:	CTGGCTTTGTCTTTCTTGTT
IL-1β Forward:	ATGAAAGACGGCACACCCAC
IL-1β Reverse:	GCTTGTGCTCTGCTTGTGAG
IL-4β Forward	ACGGAGATGGATGTGCCAAAC
IL-4β Reverse:	AGCACCTTGGAAGCCCTACAGA
IL-10β Forward:	CCTGGTAGAAGTGATGCCCC
IL-10β Reverse:	GATGCCGGGTGGTTCAATTT
IL-13β Forward:	TGACCAAGTTCTCTTCGTTGACAA
IL-13β Reverse:	CACAGCCAGTCCTCTTACTTCAC
CCL2 Forward:	TGCCCTAAGGTCTTCAGCAC
CCL2 Reverse:	ACTGTCACACTGGTCACTCC
CX3CL1 Forward:	GGCTACTGGCTTTCCTTGGT
CX3CL1 Reverse:	TAGCGGAGGCCTTCTACCAT

### Enzyme-linked immunosorbent assay (ELISA)

Inflammatory cytokine levels in the cell supernatant were measured using ELISA. The specific steps were conducted according to the manufacturer’s instructions.

### Western blotting

Brain tissues or cultured cells were lysed with cold RIPA lysis buffer and centrifuged. The supernatant was collected. Protein concentration was determined using a BCA protein assay kit. Samples were boiled with 5 × loading buffer, separated via SDS–PAGE, transferred onto a polyvinylidene difluoride (PVDF) membrane, blocked with 5% skim milk for 2 h, and then incubated with the corresponding primary antibody overnight at 4 °C. The primary antibody used was Iba-1 (1:1000). The next day, the membrane was incubated with the secondary antibody and visualized using an ECL detection reagent in a chemiluminescence imaging system. Images were saved for analysis.

### Statistical analysis

All data are represented as mean ± SD. Statistical analysis was performed using GraphPad Prism 8.0 software. One-way analysis of variance and Dunnett’s multiple comparison test were performed. A *P*-value of < 0.05 was considered statistically significant.

## Data Availability

The authors confirm that the data supporting the findings of this study are available within the article and its supplementary materials.
